# Oral Health and Psychosocial Predictors of Quality of Life and General Well-Being among Adolescents in Lesotho, Southern Africa

**DOI:** 10.3390/children8070582

**Published:** 2021-07-07

**Authors:** Abbas Jessani, Jonghm Choi, Abdul El-Rabbany, Pulane Lefoka, Mir Faeq Ali Quadri, Denise M. Laronde

**Affiliations:** 1 Schulich School of Medicine and Dentistry, University of Western Ontario, London, ON N6A 3K7, Canada; 2 College of Dentistry, University of Saskatchewan, Saskatoon, SK S7N 5E4, Canada; joc428@mail.usask.ca (J.C.); abdul.elrabbany@usask.ca (A.E.-R.); 3 Faculty of Health Sciences, Nursing Department, National University of Lesotho, Maseru 180, Lesotho; pjlefoka@gmail.com; 4 Dental Public Health, Department of Preventive Dental Sciences, College of Dentistry, Jazan University, Jizan 45142, Saudi Arabia; dr.faeq.quadri@gmail.com; 5 Oral Biological and Medical Sciences, Faculty of Dentistry, University of British Columbia, Vancouver, BC V6T 1Z3, Canada; dlaronde@dentistry.ubc.ca

**Keywords:** oral health, quality of life, adolescents, general health

## Abstract

Background: Adolescents’ quality of life is reported to be significantly associated with physical and social wellbeing. Although adolescents are 30% of the Southern African population, no previous studies have focused on this group in relation to oral health and quality of life. Methods: A 40-item survey and clinical oral examinations were conducted in public schools in Maseru from 10 to 25 August 2016. Simple, bivariate, and multivariate regressions were used to evaluate the associations of oral health and psychosocial factors with self-reported general health status and quality of life. Results: A total of 526 participants, aged 12–19 years old, responded to the survey and participated in the clinical examinations. The majority reported a good (good/very good/excellent) quality of life (84%) and general health (81%). Bivariate results showed that self-reported general health in this population was significantly influenced by age. The presence of toothache and sensitivity in the adolescents were significantly associated with poor (fair/poor) self-reported general health and were found to be the best predictors for self-general health and quality of life. Conclusions: The absence of dental conditions such as toothache and tooth sensitivity can lead to a better perception of general health and Quality of Life in adolescents.

## 1. Introduction

General health in adolescents can be viewed as a combination of a number of factors, including physical health; the absence of systemic and local disease; and mental, social, and personal well-being [1]. Although the period of adolescence typically shows the most optimal health over the course of our lives, ill-health in this period still accounts for 35% of the global burden of disease, predominantly obesity and poor mental health [2]. Many aspects of health can be affected by how adolescents take care of themselves and, in many cases, health patterns are established and track into adulthood [3,4].

General health is greatly related to the quality of life (QoL); QoL is typically used as a measure of life satisfaction. Adolescent QoL is reported to be significantly associated with physical and social wellbeing [5]. There are several factors associated with QoL. Socioeconomic status and family-related self-concept appear to be major predictors of better QoL in adolescents [6]. Similarly, age has also been described as a significant factor that influences the QoL of adolescents [7], showing a general trend of a decrease in life satisfaction as children progress through adolescence [8]. QoL also affects general health, with young children often reporting low levels of physical morbidity, and a progressive deterioration in self-rated health during the adolescent years has found to be typical [9].

Oral health has been reported to have a substantial impact on general health and QoL as it can be the source of considerable pain and suffering, and can alter eating habits, speech, and social interactions [10]. In adolescents, severe dental decays can greatly decrease their QoL, as dental caries can cause pain, discomfort, disfigurement, chronic infections, and eating and sleep disruption [11]. This results in an increased risk of hospitalization, loss of school days, a deleterious effect on nutrition and growth, and weight gain [11]. Oral health in developing countries like Lesotho is often last on the list of challenges that need to be addressed due to limited resources, poverty, and limited access to health care services [12,13]. Despite governmental efforts to provide universal primary health care for their citizens, Lesotho is still facing some of the worst health outcomes, particularly in controlling infectious diseases such as HIV/AIDS and tuberculosis [14]. The oral health status of these citizens is following a similar trend, where the most commonly reported treatments for dental caries are tooth extraction or referrals [15]. Although adolescents make up 30% of the Southern African population, no previous studies in this area have focused on adolescents as a studied and observed group [16,17]. Hence, we used the Andersen & Newman (A&N) framework to aid in the identification of the psychosocial and oral health predictors of general health and QoL of adolescents in Lesotho, Southern Africa [18]. Three broad categories are listed in the A&N framework for categorizing psychosocial factors: predisposing, enabling, and need factors. It is with the help of this framework that understanding the propensity of a population’s access to available dental services in order to satisfy their unmet dental treatment needs becomes possible [19,20,21,22].

## 2. Materials and Methods

Ethical approval was provided by the University of Saskatchewan Behavioural Ethics board (Bio-ID 650, approved 8 January 2019) for the data analysis and knowledge dissemination. This project was a joint collaboration between the Smile Lesotho Foundation (SLF), the National University of Lesotho (NUL), and the University of British Columbia (UBC). The development and execution of this project involved the collaborative participation of faculty members from academic institutions, nursing students, community dental clinicians, educational specialists, and the Ministries of Health and Education in Lesotho. As a part of the bigger project, only some aspects of the gathered data are presented in this manuscript. Refer to Jessani et al., 2021 for the detailed methodology and data collection process [23].

### 2.1. Participant Recruitment and Data Collection

The Canadian Oral Health Measure Survey and World Health Organization (WHO) household questionnaire was utilized for data collection [24,25]. The inclusion criteria included (1) adolescents enrolled in schools from grades A/6 to E/12 and (2) who submitted a signed consent form to be enrolled in the study. All the participants who did not provide a signed consent form from their guardians were excluded from this study. Adolescents were recruited through convenience sampling from ten public schools in Lesotho. Approximately 50 adolescents from each of ten schools were randomly selected to take a part in this study. After receiving consent from the guardians of the selected adolescents, the guardians completed the first half of the survey. The first half of the survey included questions pertaining to psychosocial and environmental factors, such as income and access to dental care. The participating adolescents completed the second half of the survey, which included self-reported oral health data. This questionnaire was translated into the local language of Sesotho and was completed with the assistance of local nursing students. Subsequent clinical examinations by four calibrated dentists were performed to evaluate the Decayed Missing Filled Teeth (DMFT) status of the selected adolescents. For clinical examinations, four permanent molars (molars 16, 26, 36 and 46) were examined to ascertain the DMF status. These examinations were conducted in available spaces such as classrooms, libraries, and/or playgrounds. Disposable sundries, including dental mirrors, tongue depressor, cotton roles, and magnification loupes, were utilized for clinical examinations. Oral health education sessions were conducted in each high school and all the participants were provided with oral hygiene products [23].

### 2.2. Variable Construction

The outcome variables in this study were grouped into two categories as follows:

(1) Self-reported general health, with ‘0′ being indicative of ‘excellent or very good or good’ and ‘1′ being indicative of ‘fair or poor’;
(2) Self-reported QoL, with ‘0′ being indicative of ‘excellent or very good or good’ and ‘1′ being indicative of ‘fair or poor’.

The independent variables were grouped into three categories as follows:

(1) Predisposing factors, which included age, gender, and access to oral health education.
(2) Enabling factors, which included the availability of a regular dentist and a medical doctor, having dental insurance, avoidance of dental treatment due to cost, availability of social support, and availability of dental services sought.
(3) Need factors, which included experiencing clinical dental conditions such as toothache, tooth sensitivity, bleeding when brushing, and decay, as well as satisfaction with the overall appearance of the dentition, self-reported QoL, general health, and the importance of oral health.

### 2.3. Statistical Analysis

Univariate and bivariate analyses were performed to report the strongest independent variables that had significant relationships with the outcome variables. Chi-squared tests were utilized to identify the independent factors associated with self-reported general health and QoL. Multiple logistic regressions were performed to identify the most significant factors for self-reported general health and QoL. All univariate factors with *p* < 0.10 were further assessed in the multivariable model. This analysis identified the independent variables that were statistically significant among the dependent variables in a model adjusted for other covariates. The adjusted odds ratio (OR) with a 95% confidence interval (CI) was reported and the variables with *p*-values < 0.05 were considered to be statistically significant. Statistical analysis was performed with SPSS, version 26 (SPSS Institute Inc., Cary, NC, USA). The power of the study was recalculated after completing the analyses in order to substantiate the validity of the findings. With 30% of children having toothache and poor quality of life, the prevalence coverage ratio of 0.43 and a precision of 0.05, the power of the study was estimated to be 98%. Missing data were replaced with the overall mean or median of that variable. Forty items were analyzed for the purpose of this manuscript, whereas other important findings are presented in Jessani et al., 2021 [23].

## 3. Results

A total of five hundred and twenty-six adolescents and their guardians responded to the survey and the adolescents subsequently participated in clinical examinations. Table 1 shows the gender and age breakdown of the participants. Amongst the participants, enabling factors such as the availability of a medical doctor (*n* = 42, 8%), dental insurance (*n* = 31, 6%), the ability to afford dental care (*n* = 37, 7%), and access to dental care (*n* = 74, 14%) were severely limited. However, need factors such as the presence of plaque (*n* = 316, 60%), toothache (*n* = 111, 21%), tooth sensitivity (*n* = 248, 47%), dental decay (*n* = 609, 29%), and happiness with the appearance of their teeth (21%) were notable (Table 2). Although the majority of the adolescents reported brushing their teeth at least once a day (93%) and that oral health was important (99%), 70% of the them had never been to a dentist.

Table 3 shows data representing the effect of adolescents’ predisposing, enabling, and oral health need factors on their self-reported general health and QoL. Younger adolescents (12–18 years of age) reported good or better self-reported general health (*p* = 0.006) and QoL (*p* = 0.011). Gender and access to oral health education were not found to affect either self-reported general health or QoL. None of the enabling factors affected either outcome.

However, some oral health need factors were found to influence the adolescents’ self-reported general health and QoL. Table 4 shows that adolescents who had experienced dental pain had a lower self-reported general health or QoL. Adolescents who reported toothache were more likely to report a fair or poor general health (*p* < 0.001) or a fair or poor QoL (*p* < 0.001). Tooth sensitivity also resulted in a greater proportion of fair or poor reporting for self-reported general health (*p* = 0.038) and QoL (*p* = 0.005). Not surprisingly, adolescents who were unhappy with the appearance of their teeth self-reported fair or poor general health (*p* < 0.001) and QoL (*p* = 0.002). Decay in any of the first molars did not affect self-reported general health or QoL (Table 4).

In Table 5, multiple logistic regression was adopted to report the adjusted odds ra-tio and to identify the most important predictors from A&N framework for predicting fair/poor general health and fair/poor quality of life. After the adjustment, the most important predictor for fair/poor general health was tooth sensitivity. Adolescents with tooth sensitivity were two times more likely to report fair/poor general health, compared to those without tooth sensitivity (OR: 2.22; 95% CI: 1.18, 4.17). Toothache was found to be the most significant predictor for self-reported fair/poor quality of life. Adolescents with toothache were two times more likely to report fair/poor quality of life, compared to those without toothache (OR: 2.01; 95% CI: 1.80, 3.91).

## 4. Discussion

There is a great need for dental services in the adolescent population in Lesotho and this need is reflected in their self-reported general health and QoL. We used the A&N framework of health service utilization to analyze the contributing factors. The A&N model has been used broadly across a range of marginalized populations, such as people living with HIV (PLHIV), immigrants and refugees, adolescents, homeless veterans, and prison inmates [19,21,22,26]. In our study population, this framework predicted various psychosocial and oral health factors that were associated with the self-reported general well-being and QoL of adolescents in the kingdom of Lesotho.

Approximately two thirds of the adolescents were females, and the vast majority identified their method of transportation to school was walking, as commonly reported in other African countries, such as Gambia and Malawi [27,28]. The majority of the guardians reported having no dental insurance and were not able to afford dental treatment. Similar findings were reported by Petsos, who found that the access to dental care was affected by not having dental insurance [29]. It has been found previously that people with dental insurance and those who can afford dental care have better access to dental care services and have increased utilization [21,26,29]. A little more than one quarter of the adolescents reported brushing their teeth at least once a day. This is concerning, as the average age of these adolescents was 16 years. Between the age of 10 and 24 years is an important timespan for adolescents as the behaviors and habits they develop during this time can last for their entire lives [30]. The findings that almost three quarters of the adolescents are not brushing their teeth at least once a day is worrisome as poor oral hygiene can be a strong contributor of dental decay and other oral conditions [30]. Not surprisingly, at least one third of the adolescents had decay in one or more molars, and the number of missing teeth was higher than the filled teeth. This shows that this population has limited access to both preventive dental education and services. According to an epidemiological survey in Lesotho, the preferred treatment of dental decay was extraction and our study confirmed this result.

In predisposing factors, young age was significantly correlated with both ‘excellent, or very good, or good’ general health and QoL. A study conducted by Kozmhinsky and colleagues reported a similar correlation of younger age with better self-reported QoL [31]. This could be due to one’s perception of general health and QoL changing as we age; there are various psychosocial and health-related conditions, including oral health conditions such as tooth decay, tooth sensitivity, teeth malalignment, etc., that could affect one’s perception of general health and QoL [32,33].

In oral health need factors, ‘excellent or very good or good’ general health and QoL were reported in the absence of toothache and tooth sensitivity. Having dental conditions such as toothache and tooth sensitivity can negatively affect one’s perception of general health and QoL [31,32,33]. This further confirms the synchronistic relationship between the mouth and the body [3]. Studies have shown that having a toothache and other oral conditions can have a negative impact on the daily activities of adolescents, including educational and sports-related performance, leading to dissatisfaction with self-perceived general health and QoL [34,35]. Our study also showed that the degree of satisfaction with the appearance of their teeth was directly correlated with self-reported general health and QoL. Moreover, the perception of our teeth determining our ‘self-image’ plays a significant role in our social interactions and relations [34]. According to Wilson and Cleary, the outcomes of health-related quality of life (HRQoL) experienced by an individual are not solely determined by the nature and severity of the disease/disorder but also by the characteristics of the individual and the physical and social environment, such as the appearance of the teeth [36]. Other studies revealed evidence of a positive link between self-esteem and the oral HRQoL of children and preadolescents [37,38,39,40].

Our multivariate analysis confirmed that adolescents who had reported toothache and tooth sensitivity also had two times greater odds of reporting fair/poor general health and QoL than adolescents who did not suffer these oral conditions. This substantiates the importance of maintaining good oral health by addressing acute oral conditions, such as tooth sensitivity and toothache [41]. This finding also emphasizes the importance of access to early oral health education and preventive dental care [41]. By educating adolescents about oral health care and providing preventative oral health services such as fissure sealant programs, conditions causing tooth ache and sensitivity can be greatly prevented [36,38,42,43].

Due to the convenience sampling approach, only a small percentage of adolescents per school were included in our data collection; hence, we cannot generalize the results. Not all the parents/guardians fully completed the surveys; therefore, the results may be biased. Biases within the implications of the findings may be present due to dental decay not having been examined on the full dentition. In addition, the dental decay may have been underestimated as radiographic examinations were not performed. Due to the time and logistical constrains, we were unable to fully utilize a QoL tool for the self-reported assessments. Regardless of some of the aforementioned limitations, this study provides valuable information on the self-reported general health and QoL of adolescents in Maseru, Lesotho, and their associated psychosocial and oral health predictors.

## 5. Conclusions

We found significant associations of A&N psychosocial factors and oral health predictors with self-reported oral health and self-reported QoL. For instance, self-reported oral health and self-reported QoL were associated with psychosocial factors and oral health predictors, such as predisposing factors (age, gender, and access to oral health education), enabling factors (availability of dental treatment, access to medical doctor, having dental insurance, etc.), and need factors (plaque status, toothache, etc.) as defined by the A&N framework. Additionally, we identified that the adolescents in our study face several oral-health-related challenges, including a lack of resources and dental education. A better perception of general health and QoL was found in adolescents in this population in those who had not experienced dental conditions such as toothache and sensitivity.

## Figures and Tables

**Table 1:** Predisposing and enabling factors according to the A&N framework of health service utilization (*N* = 526).

Predisposing and Enabling Factors	*N* (%)
Gender (*N* = 519)	
Male	164 (32)
Female	355 (68)
Age (years) (*N* = 523)	
12–18	468 (90)
19+	55 (10)
Access to oral health education (*N* = 518)	
Yes	105 (20)
No	413 (80)
Dental insurance (*N* = 509)	
Yes	31 (6)
No	396 (78)
Do not know	82 (16)
Availability of doctor (*N* = 511)	
Yes	42 (8)
No	469 (92)
Avoidance of dental treatment due to cost in past year (*N* = 519)	
Yes	477 (92)
No	37 (7)
Other	5 (1)
Difficulty accessing dental care when needed (*N* = 509)	
Yes	435 (86)
No	74 (14)
Mode of transportation (*N* = 316)	
Family car	2 (1)
Public transit	296 (96)
Walk/horse/others	18 (5)
School transport (*N* = 319)	
Family car	1 (0.3)
Public transit	37 (11)
Walk	281 (87)

**Table 2:** Need factors according to the A&N framework of health service utilization (*N* = 526).

Need Factors		*N* (%)	Need Factors		*N* (%)
Plaque status (*N* = 524)			Plaque status (*N* = 524)		
Absent		208 (40)	Absent		208 (39.7)
Present		316 (60)	Mild		233 (44.5)
Toothache (*N* = 523)			Moderate		70 (13.5)
No		412 (79)	Severe		13 (2.5)
Yes		111 (21)	Unhappy with appearance of teeth (*N* = 523)		
Bleeding when brushing (*N* = 523)			No		413 (79)
No		314 (60)	Yes		110 (21)
Yes		209 (40)	Sensitivity to hot/cold (*N* = 523)		
Water Fluoridation (*N* = 515)			No		275 (53)
No		223 (43)	Yes		248 (47)
Yes		162 (31)	Importance of oral health (*N* = 502)		
Do not know		130 (25)	Very or somewhat important		497 (99)
Self-reported brushing frequency (*N* = 506)			Not important		5 (1)
Never		6 (1)	Self-reported last dental visit (*N* = 517)		
Once/day		191 (38)	Less than 2 years		73 (14)
Twice/day		277 (55)	2–5 years		38 (7)
At each meal		32 (6)	More than 5 years		43 (8)
			Never		363(70)
**DMFT**			***N*** **(%)**		
	Total (*N* = 2083)	Tooth #16 (*N* = 524)	Tooth #26 (*N* = 519)	Tooth #36 (*N* = 519)	Tooth # 46 (*N* = 521)
Sound	1443 (69)	380 (72)	381 (73)	330 (64)	352 (68)
Decay	609 (29)	141 (27)	132 (26)	179 (35)	157 (30)
Missing	26 (1)	3 (1)	5 (1)	8 (1)	10 (2)
Filled	5 (0.002)	0 (0)	1 (0.2)	2 (0.4)	2 (0.4)

**Table 3:** Frequency distribution of the Andersen and Newman (A&N) predisposing and enabling factors between self-reported general health and self-reported quality of life.

Predisposing Factors	Self-Reported General Health			Self-Reported Quality of Life		
	Excellent/Very Good/Good *N* (%) *n* = 419	Fair/Poor *N* (%) *n* = 100	*p*-Value	Excellent/Very Good/Good *N* (%) *n* = 434	Fair/Poor *N* (%) *n* = 84	*p*-Value
Age						
12–18	382 (83)	81 (17)	0.006	394 (85)	68 (15)	0.011
19+	35 (35)	65 (65)		39 (71)	16 (29)	
Gender						
Male	130 (80)	33 (20)	0.811	130 (80)	33 (20)	0.124
Female	285 (81)	67 (19)		300 (86)	51 (15)	
Oral Health Education						
No	336 (82)	73 (18)	0.166	341 (84)	67 (16)	1
Yes	80 (76)	25 (24)		88 (84)	17 (16)	
**Enabling Factors**	**Self-Reported General Health**			**Self-Reported Quality of Life**		
	**Excellent/Very Good/Good**	**Fair/Poor**	***p*** **-Value**	**Excellent/Very Good/Good**	**Fair/Poor**	***p*** **-Value**
Available Regular Medical Doctor						
No	384 (88)	52 (12)	0.103	393 (84)	76 (16)	1
Yes	30 (71)	12 (29)		35 (85)	6 (15)	
Available Regular Dentist						
No	408 (81)	96 (19)	0.721	421 (84)	83 (16)	0.23
Yes	10 (77)	3 (23)		12 (100)	0 (0)	
Dental Insurance						
No	313 (79)	81 (21)	0.241	328 (83)	65 (17)	0.087
Yes	24 (77)	7 (23)		22 (71)	9 (29)	
Avoiding Dental Treatment Due to Cost						
No	28 (76)	9 (24)	0.694	30 (81)	7 (19)	0.544
Yes	385 (81)	88 (19)		398 (84)	74 (16)	
Difficulty Accessing Dental Care						
Yes	356(82)	79(18)	0.336	366 (84)	68 (16)	0.311
No	57 (77)	17(23)		59 (80)	15 (20)	

**Table 4:** Frequency distribution of the A&N need factors between self-reported general health and self-reported quality of life.

Self-Reported Need Factors	Self-Reported General Health			Self-Reported Quality of Life		
	Excellent/Very Good/Good *N* (%) *n* = 419	Fair/Poor *N* (%) *n* = 100	*p*-Value	Excellent/Very Good/Good *N* (%) *n* = 434	Fair/Poor *N* (%)* n* = 84	*p*-Value
Toothache						
No	345 (85)	62 (15)	<0.001	355 (87)	51 (13)	<0.001
Yes	73 (66)	38 (34)		78 (70)	33 (30)	
Sensitivity to Hold/Cold						
No	228 (84)	43 (16)	0.038	238 (88)	32 (12)	0.005
Yes	190 (77)	57 (23)		195 (79)	52 (21)	
Bleeding Gums When Brushing						
No	245 (81)	58 (19)	0.65	264 (85)	47 (15)	0.397
Yes	164 (80)	42 (20)		169 (82)	37 (18)	
Unhappy with Teeth Appearance						
No	348 (85)	63 (15)	<0.001	354 (86)	56 (14)	0.002
Yes	70 (65)	37 (35)		79 (74)	28 (26)	
Importance of Oral Health						
Extremely important/important	404 (81)	93 (19)	0.8	416 (84)	80 (16)	1
Not important	4 (80)	1 (20)		5 (100)	0 (0)	
**Clinical Need Factors**	**Self-Reported General Health**			**Self-Reported Quality of Life**		
	**Excellent/Very Good/Good** ***N*** **(%)** ***n*** **= 419**	**Fair/Poor** ***N*** **(%)** ***n*** **= 100**	***p*** **-Value**	**Excellent/Very Good/Good** ***N*** **(%)** ***n*** **= 434**	**Fair/Poor** ***N*** ** (%)** ***n*** **= 84**	***p*** **-Value**
Tooth Decay # 16						
Sound	306 (82)	69 (18)	0.454	318 (85)	56 (15)	0.282
Decayed	111 (79)	30 (21)		114 (81)	27 (19)	
Tooth Decay #26						
Sound	311 (82)	66 (18)	0.244	314 (84)	62 (16)	0.679
Decayed	102 (78)	29 (22)		112 (85)	19 (15)	
Tooth Decay # 36						
Sound	267 (82)	58 (18)	0.29	277 (85)	47 (15)	0.37
Decayed	140 (78)	39 (22)		147 (82)	32 (18)	
Tooth Decay #46						
Sound	286 (82)	61 (18)	0.388	293 (85)	53 (15)	0.792
Decayed	124 (79)	33 (21)		131 (83)	26 (17)	
Plaque status						
Absent	238 (88.1)	33 (11.9)	0.98	200 (85.5)	34 (14.5)	0.11
Present	170 (78.7)	46 (21.3)		140 (73.7)	50 (26.3)	

**Table 5:** Multiple logistic regression reporting the most important predictor for fair/poor general health and fair/poor quality of life.

Factors	Self-Reported General Health				Self-Reported Quality of Life			
	Unadjusted OR (95%CI)	*p*-Value	Adjusted OR ^1^ (95%CI)	*p*-Value	Unadjusted OR (95%CI)	*p*-Value	Adjusted OR ^1^ (95%CI)	*p*-Value
Age	8.78 (5.48, 14.05)	<0.001	-	NS	-	-	-	-
Toothache	2.90 (1.80, 4.66)	<0.001	-	NS	2.94 (1.78, 4.86)	<0.001	2.01 (1.80, 3.91)	<0.001
Sensitivity	1.59 (1.02, 2.47)	0.04	2.22 (1.18, 4.17)	0.01	1.98 (1.23, 3.20)	0.005	-	NS
Unhappy with Tooth Appearance	2.92 (1.81, 4.72)	<0.001	-	NS	2.24 (1.34, 3.75)	0.002	-	NS

^1^ Stepwise (backward) logistic regression. NS: not significant.
